# Cancer in the Elderly

**DOI:** 10.1155/2014/872029

**Published:** 2014-04-30

**Authors:** Frank Buntinx, Christine Campbell, Marjan van den Akker

**Affiliations:** ^1^Department of General Practice, Catholic University of Leuven, Leuven, Belgium; ^2^Department of Family Medicine, Research School Caphri, Maastricht University, Maastricht, The Netherlands; ^3^Centre for Population Health Sciences, The University of Edinburgh, Medical Quad, Teviot Place, Edinburgh EH8 9AG, UK

The incidence of cancer rises among older populations [1, 2], and continuous improvement in treatment outcomes is resulting in even greater increases in the prevalence of cancer survivors in this age group [3]. There is considerable variation in cancer mortality rates in the elderly among high-income countries, with the UK having poorer outcomes compared to the USA and Western and Northern European countries [4]. The diagnosis of cancer and treatment decisions following diagnosis at an older age bring specific challenges to health care providers. Further, living with cancer has specific characteristics and consequences for older people. The five papers (three from North America, two from Europe) included in this special issue address some of these topics: increasing awareness of breast cancer symptoms, management of patients with lung and breast cancers, and survivorship issues specific to older patients.

There is good evidence that age is a risk factor for the delay in presentation with breast cancer [5]. L. J. L. Forbes et al. describe the implementation into routine clinical practice of an evidence-based brief intervention designed to promote earlier symptomatic presentation of breast cancer among older women. The authors have previously reported on the effectiveness of the intervention in a randomised control trial [6]; this further work shows that its introduction into routine mammography appointments at four pilot areas within the UK's Breast Screening Programme results in similar levels of breast cancer awareness among participating women (mean age 71 years, 4 months) as in the trial setting. The intervention was acceptable to women and to mammography providers. Given the low awareness of age-related cancer risk within the UK compared to a number of other countries [7], interventions of this nature, conveying key cancer messages as patients are entering the age group with highest risk of breast cancer, have the potential to contribute to earlier health seeking.

Subsequent to a cancer diagnosis, treatment decisions for older patients are often complicated by factors such as frailty, and the presence of comorbidities. M. K. Malik et al. have examined the impact of treatment decisions among women aged 71 and over with a breast cancer diagnosis compared to younger women using a retrospective observational study design in a population of patients receiving potentially curable surgery. Patients were from two health care facilities in NY, USA. The results include differing pathologies between younger and older women and significant differences in proportion of patients given adjuvant or neoadjuvant chemotherapy and radiation therapy. However, among this patient group, undertreatment (defined as lack of adherence to conventional treatment guidelines) did not lead to poorer local or distant disease-free survival compared to appropriately treated individuals. Given the selected population in this study, the authors emphasise the need for optimal treatment regimens to be determined on a case-by-case basis.

Despite legitimate concerns about the ability of some older patients to tolerate aggressive treatments, S. Fisher et al. demonstrate that there are elderly patients who do receive a survival benefit from chemotherapy for small cell lung cancer (SCLC), even at reduced doses. They assessed the uptake and tolerance of chemotherapy among patients aged 75 and older with SCLC in AB, Canada. 68% of patients who were recommended chemotherapy by an oncologist began treatment: 52% completed all cycles, with 41% receiving reduced chemotherapy doses. Kaplan-Maier survival curves show that patients who completed chemotherapy had a significantly better survival than those who did not, and Cox adjusted hazard ratios show this benefit existed even when the chemotherapy dose was reduced. The authors suggest that elderly patients are at least considered for established treatments, with further research needed into the relationship between frailty and toxicity to help determine who might benefit from chemotherapy treatment.

With an increasing number of cancer diagnoses and improved outcomes, the number of older cancer survivors is increasing. Patients (and their health care providers) must manage not only the sequelae of treatment but also the increasing burden of morbidity experienced with older age. The paper by L. Deckx et al. compares the chronic disease burden among cancer survivors aged 60 years and older with up to four controls matched for age, sex, and general practice, all drawn from a primary care database in the Netherlands. The results from this retrospective cohort study indicate similarly high levels of chronic disease among cancer patients prior to their diagnosis when compared with noncancer patients. The most common preexisting chronic diseases included diabetes, lipid disorders, ischaemic heart disease, and myocardial infarction, with only chronic obstructive pulmonary disease (COPD) significantly more prevalent among lung cancer patients. Among cancer survivors and noncancer patients, the incidence of chronic disease was again similar; venous thrombosis was more common in the two years after diagnosis in cancer survivors. Given their experience and expertise in managing multimorbidity, the authors emphasise the important role that general practitioners can have in supporting cancer survivors.

K. M. Bellizi et al. further examine the impact of age among cancer survivors in CA, USA, but extend the analysis to include the impact of race/ethnicity on health-related quality of life. The population-based questionnaire survey among adult survivors of breast, prostate, colorectal, ovarian, or endometrial cancer examined physical and mental function by age, ethnicity/race, and type of cancer, as well as potential interactions. The authors describe a double jeopardy in their study population, where a significant interaction effect between age and race/ethnicity impacting physical function is observed, persisting among older males with prostate cancer even after controlling for comorbidity. This is a salient reminder that not all older patients with cancer are alike in sociodemographic factors which may have had a profound effect on health status and on earlier stages of a patient's cancer journey are likely to continue to impact on health outcomes in the survivorship phase too.

Together, these papers highlight a number of important issues. Who are the elderly? There is no common definition across the papers with cutoffs of 60, 65, 70, 71 and, 75 years being used. These choices are largely pragmatic, reflecting the data sources available or the population in whom an intervention was being tested. However, it needs to be remembered that there are important differences within the “elderly.” Not surprisingly, different responses to treatment are observed, increasing morbidity with age, and as noted above sociodemographic factors that shape the context of people's living experience remain important.

The importance of comorbidity and indeed multimorbidity comes through clearly with respect to treatment decisions and outcomes. Not all patients will benefit from treatment due to these other concomitant illnesses, or they may have less resilience to side effects, adversely impacting on quality of life. For patients who may already have a limited quality of life, the decision to undergo treatment is one that requires both clinical judgment and consideration of patient (and perhaps caregiver) preferences as well as contextual factors [8]. Good examples of such an approach exist, for example, [9]. Ideally, a multidisciplinary approach, including where appropriate the oncologist, general practitioner, geriatrician, cancer nurse specialist, and possibly the palliative care team, as well as the patient and family, will be adopted.

What should be the ongoing research agenda for this growing and challenging patient population? All aspects of the cancer control continuum (prevention, screening, detection and diagnosis, treatment, and survivorship) are relevant to older as well as younger patients, but the influence of age on many of these is still poorly understood.

Further work to identify which older patients might benefit from specific forms of cancer screening is needed [10], although the role of the general practitioner in this decision-making process is known to be important [11]. The influence of age and associated morbidities on the diagnostic accuracy of signs and symptoms or of diagnostic algorithms for specific cancer types and any subsequent differences in the diagnostic pathways in the elderly compared to those in younger cancer patients (including the impact, if any, of these differences) on the time of diagnosis and commencement of treatment requires further elucidation.

Further research is also needed on the role of patient preferences in determining treatment strategies following a diagnosis of cancer, optimal modes of information provision, and understanding determinants of patient suitability (physical and psychological) in the selection of appropriate therapy (whether chemotherapy, radiotherapy, surgery, and nonaggressive management). Better data on comparative outcomes of chemotherapy regimens, radiotherapy, and surgery between younger and older patients to guide both patients and providers is needed. Other areas meriting investigation include the effect of comorbidity on the response to treatment in elderly patients and the impact of a cancer diagnosis (and treatment) on psychological outcomes in elderly patients compared to younger patients.

The need for greater involvement of older people in cancer clinical trials has been recognised [12], but there is also a need for other research designs including qualitative ones where the voices of older people themselves—their attitudes towards health and treatment decisions—are heard.

Although not dealt with in this special issue, it is important too to remember the international context of the growing global burden of cancer among the elderly. More than half of new cancer diagnosis already occurs in less developed regions of the world; demographic changes including increasing life expectancy in many low- and middle-income countries will result in cancer (as well as other noncommunicable diseases) giving rise to considerable health care challenges in the older population in this century. How cancer services are developed to address these is of growing concern [13, 14].

*Frank Buntinx* *Frank Buntinx* *Christine Campbell* *Christine Campbell* *Marjan van den Akker* *Marjan van den Akker*
